# Nonlinear Lifespan Trajectories of Memory and Fluid Reasoning: A Longitudinal GAMM Study

**DOI:** 10.21203/rs.3.rs-8313556/v1

**Published:** 2025-12-11

**Authors:** Georgette Argiris, Yaakov Stern, Christian Habeck

**Affiliations:** a Cognitive Neuroscience Division, Department of Neurology, Columbia University Irving Medical Center, New York, NY; b Taub Institute, Columbia University, New York, NY

**Keywords:** cognitive aging, RANN, GAMM, memory, fluid reasoning, longitudinal, posterior association cortex

## Abstract

Longitudinal studies in cognitive aging provide critical advantages over cross-sectional designs, with growing consensus that flexible nonlinear approaches are needed to capture the complexity and heterogeneity of lifespan neural change. In the present study, we leveraged three waves of fMRI data and implemented generalized additive mixed models (GAMMs) to characterize linear and nonlinear trajectories of brain activation across the cognitive domains of memory and fluid reasoning, and to identify regions displaying age-varying cognition–brain coupling. Participants (memory: N = 431; fluid reasoning: N = 441; ages 20–80+) from the Reference Ability Neural Network study completed up to three fMRI assessments over 0–12 years. Domain-level activation maps were parcellated using the Glasser atlas and modeled with tensor-product smooths of baseline age and time, alongside age-varying coefficient terms linking within-person activation fluctuations to in-task performance. Cognition-only models showed robust, approximately monotonic age-related declines in both domains, with no reliable Age × Time interaction. In contrast, activation models revealed pronounced nonlinear Age × Time effects concentrated almost exclusively in posterior association cortex. A particularly notable example was the right POS2 (precuneus/cuneus), which exhibited a midlife reduction in activation, consistent with evidence implicating the precuneus as an early site of functional vulnerability in aging. Beyond these activation trajectories, cognition–activation coupling analyses uncovered age-varying relationships across posterior midline and dorsal parietal regions as well as left rostral Area 6 and IFJp, including several age-dependent changes in the strength and sign of coupling. Posterior midline regions shifted from positive to weaker or negative coupling with age, whereas left rostral Area 6 and IFJp showed the inverse pattern, with negative coupling in youth that diminished or reversed in older adulthood. Together, these findings highlight posterior association cortex as a major site of functional reorganization across the adult lifespan and demonstrate that flexible GAMM approaches reveal developmental inflection points and shifts in brain–behavior coupling that remain undetectable using linear or cross-sectional methods.

## 1. Introduction

Cognitive functions and their neural substrates change dynamically across the adult lifespan (Gazes et al., 2021; Rieckmann et al., 2017; Hartshorne & Germine, 2015; Hakun et al., 2015; for review, see Grady et al., 2012). Hartshorne and Germine (2015), for instance, demonstrated that different cognitive abilities peak and decline at varying points throughout adulthood, revealing considerable heterogeneity and dynamic change in cognitive performance across the lifespan. Longitudinal studies in cognitive aging provide critical advantages over cross-sectional designs. While cross-sectional studies compare different age groups at a single time point and are susceptible to cohort effects, practice effects, and individual baseline differences, longitudinal studies track within-person changes over time, offering a more accurate depiction of age-related cognitive and brain changes. A recent review by Kang and colleagues (2024) synthesized data from 63 longitudinal and cross-sectional MRI studies and demonstrated that longitudinal study designs systematically yield larger standardized effect sizes and greater replicability in brain-wide association studies compared to cross-sectional designs.

Both linear and nonlinear trajectories of cognitive aging have been reported in the literature, with evidence for nearly linear declines in some domains (e.g., processing speed) and accelerating, nonlinear declines in others (e.g., memory and fluid reasoning; LaPlume et al., 2022; Salthouse, 2019; Verhaeghen, P., & Salthouse, T., 1997). Recent large-scale and longitudinal studies confirm that cognitive performance often shows gradual decline in early and mid-adulthood, followed by a transition to more rapid and variable decline in later life, supporting the need to move beyond simple linear models (LaPlume et al., 2022; Abbott et al., 2018). However, most research still relies on linear or quadratic models, which impose rigid assumptions about the shape of cognitive change and often fail to detect age-related shifts in decline (Salthouse, 2016; 2019), despite a growing consensus that more flexible nonlinear analyses are needed to fully capture the complexity and heterogeneity of cognitive aging (Ghisletta et al., 2020). For instance, in a comprehensive review of five years of cognitive aging research, Ghisletta and colleagues (2020) report a large bias towards linear or polynomial models, with fewer than 5% employing truly nonlinear approaches. The authors emphasize that this bias can lead to misleading conclusions, due to model misspecification and insensitivity to changes in variance, strongly encouraging the adoption of nonlinear methods to better capture true individual differences in cognitive aging.

Compared to the extensive longitudinal literature on cognitive aging, research on brainbased change is more limited. While large-scale lifespan analyses have attempted to characterize age-related brain trajectories in domains such as white-matter structural networks (Conte et al., 2024) and gray-matter–derived structural and functional network correlations (Luo et al., 2020), reporting nonlinear U-shaped patterns, these findings rely on restrictive quadratic-term modeling applied to cross-sectional data. Nonetheless, smaller-scale longitudinal structural MRI studies have begun to provide substantial insight into age-related brain changes across the lifespan. For instance, Vinke and colleagues (2018) reported predominantly nonlinear age-related trajectories across volumetric, microstructural, and focal brain measures, characterized by accelerating change with advancing age. While some cortical and subcortical regions exhibited relatively linear decline or stability across time, many regions showed nonlinear patterns, highlighting the heterogeneous and region-specific nature of structural atrophy. In line with these observations, Sørensen et al. (2021) further demonstrated that longitudinal trajectories of structural integrity vary markedly across brain regions and deviate from simple linear or uniform patterns. Using generalized additive mixed models (GAMMs) on multi-wave MRI data, they identified distinct, region-specific curves of age-related change as well as differences in the timing and rate of decline, supporting the notion that structural brain aging unfolds along diverse, nonlinear trajectories across the cortex and subcortex. Additionally, Bethlehem et al. (2022) used generalized additive models to generate normative “brain charts” across gray matter, white matter, and CSF from over 120,000 MRI scans spanning 100 years of age. Owing to a low percentage of the dataset containing longitudinal measurements, the trajectories were estimated cross-sectionally and only validated with the available longitudinal data, but still reveal clear, tissue-specific developmental and aging patterns, providing a valuable normative framework for situating individual structural profiles.

Previous work in our lab extended the “brain chart” and atlas-based perspective to functional activation to compare cross-sectional and longitudinal measurements of neural activation across the adult lifespan (Argiris et al., 2021). We utilized a Gaussian age kernel to generate continuous, age-weighted maps of change for both neural activation as well as behavioral performance, producing “templates” of change analogous to 4D MRI atlas approaches (e.g. Ericsson, Aljabar, & Rueckert, 2008). We found that the regions showing maximal age-related differences often diverged substantially between cross-sectional and longitudinal estimates, particularly in the memory and fluid reasoning domains. In contrast to the commonly reported pattern of frontal over-recruitment in cross-sectional studies, the longitudinal analyses highlighted increased activation in posterior regions among older adults, revealing trajectories that are not apparent when examining age differences at a single time point. Together, these findings underscored the potential cross-sectional mischaracterization of both magnitude and spatial distribution of age-related change and demonstrated the necessity of longitudinal approaches for capturing the dynamic, domain-specific nature of functional brain aging.

In the present study, we leverage three time points and implement GAMMs to flexibly model trajectories of brain activation in the domains of memory and fluid reasoning. GAMMs offer a flexible, data-driven method for estimating nonlinear trajectories in neural activation across the lifespan and are well suited for handling irregular measurement intervals and individual differences in aging patterns (Sørensen et al., 2021). By modeling age-varying slopes in time-related change, GAMMs will enable us to detect critical periods—such as midlife transitions—when neural activation patterns in memory and fluid reasoning may shift in direction or magnitude and additionally relate to change in cognitive trajectories. Longitudinal neuroimaging studies have primarily focused on age-related reorganization of large-scale functional networks, typically using linear mixed models to document shifts in within- and between-network segregation and integration in systems such as the frontoparietal, sensorimotor, and default mode networks (Deery et al., 2022; Oschmann et al., 2020; Staffaroni et al., 2018), with far fewer investigations characterizing age-related changes in task-evoked activation (see Jäncke et al., 2022). By applying nonlinear GAMMs to task-specific activation within a longitudinal dataset, the present study provides a first attempt at establishing flexible, lifespanspanning activation trajectories for the domains of memory and fluid reasoning, thereby addressing a critical gap in the functional aging literature.

## 2. Methods

### 2.1 Participants

**Figure 1.**
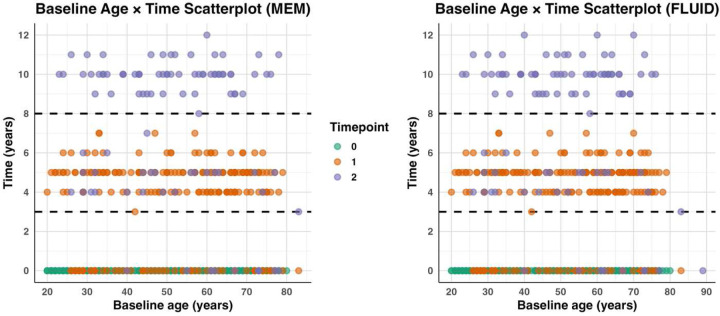
Scatterplot of baseline Age × Time for both the MEM and FLUID domain. Each dot represents a participant’s measurement data point, plotted by baseline age and elapsed time since baseline. Colors indicate the measurement wave (i.e., T0, T1, T2). Horizontal dashed lines mark the earliest observed times at which second- and third-visit measurements occur in the dataset. These plots show the sampling pattern across age and time, illustrating that participants span a wide range of baseline ages and follow-up durations, with comparable coverage across the MEM and FLUID cohorts.

Participants were drawn from the Reference Ability Neural Network (RANN) study, a community-based cohort from the greater New York area. To maximize participant inclusion, we did not limit analyses to individuals with complete data for both memory and fluid reasoning; as a result, the number of observations differed across domains. An initial sample of 433 participants contributed data to the memory domain and 443 to the fluid reasoning domain. Two memory participant and two fluid participants were excluded as systematic outliers due to multiple extreme values in their task-activation distributions. The final samples therefore consisted of 431 participants for memory and 441 for fluid reasoning. Participant counts across study waves (from one to three time points) and baseline demographics stratified by age bracket are presented in Table 1. Additionally, to characterize the longitudinal design, we display baseline age × time scatterplots depicting the distribution of observations across the three waves (with time ranging from 0 at baseline to approximately 12 years; see Figure 1). All participants were native English speaking, right-handed (Old-field Edinburgh Handedness Inventory; Oldfield, 1971) adults. Participants were recruited via random market advertising. All participants underwent screening for severe medical or psychiatric disorders, head injuries, hearing or vision impairments, and any other factors that might interfere with MRI acquisition. Older adults were additionally evaluated for dementia and mild cognitive impairment at all time points using the Dementia Rating Scale (Mattis, 1988).

### 2.2 Procedure

The experiment was designed to collect fMRI data as participants completed 12 computerized cognitive tasks linked to four reference abilities (RAs; Stern et al., 2014). Here, we will focus on the memory and fluid reasoning domains only. The study included up to three longitudinal time points (T0, T1, T2), spanning 0–12 years from baseline. At each wave, the 12 tasks were administered in two ~2-hour sessions, each containing six tasks from two RAs. Tasks within each RA followed a fixed order, while the order of the two sessions was counterbalanced across time points. Follow-up task administration was fully randomized relative to prior waves, and all tasks were identical across time points.

As the analyses were performed at the domain level, participants were required to complete only one task within a given RA to be included, allowing maximal longitudinal retention. Before each scanning session, participants completed an out-of-scanner training session on a laptop to familiarize themselves with the six tasks for that session. Training was self-paced with breaks permitted, and participants could repeat the training if needed. Task comprehension was confirmed based on participants’ reported comfort and the judgment of a trained research assistant. During scanning, breaks were allowed between the cognitive and structural scans but were rarely requested. Participants also completed a neuropsychological battery in a separate session, which is not discussed here.

#### 2.2.1 Stimulus presentation

Stimuli were backprojected onto an LCD monitor positioned at the end of the scanner bore. Participants viewed the screen via a tilted mirror system, which was mounted on the head coil. When needed, vision was corrected to normal using MR-compatible glasses (SafeVision). Responses were made on a LUMItouch response system (Photon Control). E-Prime version 2.08, operating on a PC platform, was used for stimulus delivery and data collection. Task onset was electronically synchronized with the MRI acquisition device.

#### 2.2.2 Reference ability in-scanner tasks

Twelve cognitive tasks, each belonging to one of four reference abilities, were administered in the scanner. In the present report, we focus exclusively on the memory and fluid reasoning domains, each comprising three tasks. A brief description of each task, organized by domain, is provided below (see Stern et al., 2014, for full task details). All tasks required responses via button press. For both memory and fluid reasoning tasks, accuracy—calculated as the proportion of correct trials out of all included trials—served as the primary performance measure. Accuracy scores for each task were z-score–transformed at each testing wave using fixed reference means and standard deviations derived from the baseline sample. For statistical analyses, tasks were then averaged within each reference domain to yield a single cognitive score per domain. Throughout this document, we use abbreviated labels for the two reference abilities: MEM (memory) and FLUID (fluid reasoning). We also use the terms *domain* and *reference ability* interchangeably.

##### 2.2.2.1 MEM

For all three episodic memory tasks, the study and test phases were scanned together and analyzed as a single run. Accuracy (percentage of correct trials) served as the behavioral variable. The tasks were as follows: (1) *Logical memory*—Participants read a brief story on the screen and answered detailed multiple-choice questions about its content, choosing between four possible answers; (2) *Word order recognition*—Participants studied a 12-word list presented one word at a time and, at test, identified which of four options followed a given probe word; and (3) *Paired associates*—participants studied 12 word pairs and, at test, selected which of four options had been paired with a given probe word.

##### 2.2.2.2 FLUID

Accuracy (percentage of correct trials) served as the behavioral variable. The tasks were as follows: (1) *Matrix Reasoning* (adapted from Raven, 1962)—Participants viewed a 3×3 matrix with the final cell missing and selected which of eight options best completed the pattern; (2) *Letter Sets* (Ekstrom et al., 1976)—Participants viewed five sets of letters, four following a common rule, and identified the set that violated it; and (3) *Paper Folding* (Ekstrom et al., 1976)—participants saw diagrams of a folded sheet with holes punched through it and chose which of six options depicted the correct hole pattern once unfolded.

#### 2.2.3 fMRI data acquisition

Task-based functional images were acquired across three 3T scanners from different manufacturers (Philips Achieva, Siemens Prisma, and GE SIGNA Premier). All runs were collected using gradient-echo echo-planar imaging (EPI) sequences optimized for whole-brain task fMRI. Because specific sequence parameters (i.e., TR, TE, flip angle, field-of-view, matrix and voxel size, slice number, bandwidth, and acceleration factor) necessarily varied across scanners due to manufacturer-dependent implementation constraints, full scanner-specific EPI acquisition details are provided in Supplementary Table 1 (ST1). Structural T1-weighted images were acquired for standard preprocessing steps such as co-registration and normalization but were not used in any analyses; therefore, detailed T1 acquisition parameters are not reported here. A neuroradiologist examined each participant’s scan for abnormalities, and any significant findings were reported to the participant’s primary care physician.

#### 2.2.4 fMRI data preprocessing

Preprocessing and first-level modeling were performed in FSL (version 5.7; Smith et al., 2004) and custom Python scripts. For each task fMRI run, preprocessing included (1) generation of within-participant noise histograms (FEAT); (2) motion correction via spatial realignment to the middle volume using FMRIB’s Linear Image Registration Tool (MCFLIRT); (3) slice-timing correction; (4) creation of a brain mask from the first volume; (5) high-pass filtering (128s); (6) prewhitening to attenuate temporal autocorrelation; (7) general linear model (GLM) estimation with motion-related nuisance regressors and a double-gamma HRF; and (8) nonlinear registration of functional images to the participant’s structural scan followed by normalization into MNI space using FMRIB’s Nonlinear Image Registration Tool (FNIRT). Fluid reasoning tasks, which followed a block design, were modeled using boxcar regressors convolved with the canonical HRF, with the intrinsic baseline defined as the inter-block intervals. Memory tasks were modeled using an event-related design in which the onset-to-response interval for each correct retrieval trial was convolved with the canonical HRF; although encoding, retention, and retrieval were acquired, only retrieval was analyzed. Each GLM produced a parameter-estimate (β)-map for the corresponding task. A gray-matter probability mask (≥50% mean probability across participants) was generated separately for each domain, reflecting the domain-specific sample composition, and applied to all β-maps. This resulted in 31,018 voxels for each memory domain task and 31,087 voxels for each fluid reasoning domain task. All downstream analyses were conducted on these masked first-level activation maps, which were averaged across tasks within each domain to yield a single domain-level activation map per individual.

#### 2.2.5 ROI parcellation

To limit the number of model comparisons across voxels, we applied the HCP-MMP1.0 atlas (Glasser et al., 2016) to each individual’s β-map in MNI space. Given that the original atlas is surface-based, we applied the volumetric MNI152 version distributed by AFNI (i.e., MNI_Glasser_HCP_v1.0.nii.gz; Cox, 1996; the updated atlas file was obtained from https://afni.nimh.nih.gov/pub/dist/atlases/MNI_HCP/MNI_Glasser_HCP_2019_v1.0/). We chose the HCP-MMP1.0 atlas because, unlike parcellations based solely on anatomical landmarks, it combines multiple MRI modalities (myelin maps, cortical thickness, resting-state and task fMRI, and diffusion connectivity) and uses multimodal surface matching to define functionally and microstructurally informed cortical boundaries (Glasser et al., 2016). The atlas is composed of 360 total parcels (180 parcels per hemisphere) and was resampled from 1 mm to 3 mm voxel space using a random participant’s β-map template, with nearest-neighbor interpolation applied to preserve discrete parcel labels. Due to voxel restrictions based on the union of group-level gray matter probability masks (>50%), full atlas coverage was not attainable, resulting in 354 regions for the memory domain and 356 regions for the fluid-reasoning domain. Individual ROI values were obtained by taking the median value across all voxels belonging to that ROI.

#### 2.2.6 Harmonization across scanners

We considered applying ComBat harmonization (Fortin et al., 2018; Johnson et al., 2007) to adjust for scanner-related differences across waves, but ultimately decided against it due to the substantial imbalance in scanner sample sizes in our dataset (e.g., for the fluid reasoning task: 595 Philips scans, 11 GE scans, and 108 Siemens scans, with one wave acquired almost entirely on a single scanner). Although ComBat is frequently used for multi-site harmonization, recent work indicates that its assumptions and stability issues extend to within-site scenarios where scanner models, sequence updates, or wave-specific hardware changes create batch effects (see Jodoin et al., 2025). In particular, ComBat assumes that covariate effects are consistent across scanners and that scanner effects behave as additive and multiplicative shifts around shared biological slopes. When scanner waves differ greatly in sample size or covariate distributions—as in our longitudinal design—these assumptions may be violated, leading to biased or unstable harmonization that is further exacerbated by ComBat’s empirical Bayes shrinkage pulling small-scanner estimates toward the global mean. Moreover, empirical studies suggest that ComBat requires approximately 16–32 subjects per batch for stable estimation—consistent with Orlhac et al. (2021) minimum recommendation of 20–30—and our smallest scanner wave is substantially under this threshold. Given these limitations and the complex nature of scanner effects in task-based fMRI activation data, we implemented a custom residualization approach to account for scanner-related differences in the β maps. We applied a regression-based residualization procedure separately for the memory and fluid reasoning domains, each of which had a distinct sample composition. For each domain, β maps across all available participants and time points were vectorized and stacked into a single observation vector y, and a general linear model was fit with a design matrix X = [X_scanner_, X_aging_, X_subject_]. Here, X_scanner_ contained three scanner indicators (Philips, Siemens, GE), X_aging_ encoded timepoint effects, and X_subject_ consisted of subject-specific intercepts capturing time-invariant individual differences. Scanner effects were removed by excluding the scanner columns from the reconstructed fit—i.e., setting the scanner coefficients to zero and recomputing $\hat{y}$ using only the aging and subject components and adding back the residuals. Formally, this is ${\hat{y}}_{\text{resid}}={\text{X}}_{\left(-\text{scanner}\right)}{\hat{\beta }}_{\left(-\text{scanner}\right)}+\left(y-\text{X}\hat{\beta }\right)$, and all downstream analyses were performed on these scanner-residualized β maps.

### 2.3 Generalized Additive Mixed Models (GAMMs) specification

To examine age-related differences in longitudinal outcomes, we used generalized additive mixed models (GAMMs) implemented with the gamm() function in the mgcv package in R (Wood, 2017). This approach combines GAM smoothers with mixed-effects modeling via *nlme*, allowing us to account for correlated errors (e.g., within-subject autocorrelation) and random effects while estimating nonlinear relationships. However, nlme::lme is also susceptible to convergence issues when the underlying likelihood becomes numerically indefinite, an issue encountered in approximately 6% of model cases when utilizing the default variance-component search range (mgcv.vc.logrange = 25). Therefore, following the recommendations in the mgcv documentation (mgcv package version 1.9–4; Wood, 2024), we restricted the allowable range of the log variance-component parameters by adjusting the setting option (mgcv.vc.logrange = 15). to improve stability without compromising model flexibility. This constraint substantially reduced convergence issues (to ~1.5% of models) and did not substantively alter the estimated smooth functions or fixed-effect parameters in sensitivity checks. Convergence diagnostics were tracked for all ROI-specific models, and we confirmed that all models yielding statistically significant effects converged and did not display warning flags.

We specified cubic regression splines for baseline age and time, setting *k* = 15 and *k* = 5, respectively; while Sørensen et al. (2021) used *k* = 20 for the age smooth and *k* = 5 for time, we reduced the upper-limit basis dimension for the age smooth to retain adequate nonlinear flexibility while avoiding overspecification given our smaller sample. The tensor-product interaction smooth was assigned matching marginal dimensions (*k* = c(15, 5)). Smoothing parameters were estimated via restricted maximum likelihood (REML), allowing the effective degrees of freedom (edf) for each smooth term to be penalized based on model complexity. This approach enables flexible modeling of potentially nonlinear age trajectories while appropriately controlling for inter-individual variability. To assess linearity, we used the estimated effective edf for each smooth. An edf of 1 corresponds to a strictly linear relationship, values slightly above 1 represent weak nonlinearity, and edf > 2 reflects clear nonlinearity (Zuur et al., 2009). However, these ranges are interpretive rather than prescriptive as no strict edf cutoffs exist to our knowledge; we therefore treated edf as a continuous indicator of model complexity.

#### 2.3.1 Brain Activation and Cognitive Models

For both the longitudinal brain-activation analyses and the cognitive analyses, we fit generalized additive mixed models (GAMMs) with identical predictor specifications. In the activation analyses, separate models were estimated for each region of interest (MEM: 354 ROIs; FLUID: 356 ROIs), with ROI-specific activation as the outcome. In the cognitive analyses, z-score–transformed accuracy scores were modeled separately for each cognitive domain. For participant *i* at time point *t*, the outcome variable was modeled as:

(1) $$y_{it}=\beta_{0}+f_{1}\left(Age_{i0}\right)+f_{2}\left(Time_{it}\right)+f_{3}\left(Age_{i0},Time_{it}\right)+\beta_{1}Sex_{i}+\beta_{2}Edu_{i}+b_{i}+\varepsilon_{it},$$

where *f*_1_ and *f*_2_ are smooth functions modeled using cubic regression splines, and *f*_3_ is a tensor-product smooth capturing the interaction between baseline age (Age_*i*0_) and time since baseline. Sex and years of education were included in the model as linear covariates. A subject-specific random intercept *b*_i_ accounted for individual differences in baseline levels by allowing the intercept to vary for each subject. Our main term of interest was the tensor-product interaction smooth *f*_3_(Age_*i*0_, Time_*it*_) .

#### 2.3.2 Cognitive models with ROI activation as predictor

In a second set of cognitive models, we examined whether regional brain activation predicts cognitive performance and whether this activation–cognition coupling varies as a function of baseline age. Essentially, the question that we were interested in answering is whether time-varying fluctuations in an individual’s activation are associated with corresponding fluctuations in cognition, and whether the strength of this within-person coupling differs by baseline age. To separate stable between-individual differences from within-individual change, regional activation was decomposed into an individual-specific mean (ROI_*b*_) and a within-person deviation term (ROI_*w*_). For participant *i* at time point *t*, cognition was modeled as:

(2) $$y_{it}=\beta_{0}+f_{1}\left(Age_{i0}\right)+f_{2}\left(Time_{it}\right)+f_{3}\left(Age_{i0},Time_{it}\right)+\beta_{1}Sex_{i}+\beta_{2}Edu_{i}+\beta_{3}ROI_{b,i}+f_{4}\left(Age_{i0}\right)*ROI_{w,it}+b_{i}+\varepsilon_{it},$$

where *ROI*_*b,i*_ represents a scalar value capturing the between-individual association between overall activation level and cognition, and the term *f*_4_(*Age*_*i*0_)*ROI*_*w,it*_ represents the age-varying coefficient smooth that tests whether the within-person activation–cognition coupling—that is, whether time points in which an individual shows higher activation than their own individual average are associated with higher or lower cognition—varies as a smooth function of baseline age. For the age-varying activation–cognition coupling term *f*_4_(*Age*_*i*0_)*ROI*_*w,it*_, we used a cubic regression spline with k=10. This basis dimension is lower than that used for the main age smooth because the coupling surface is a higher-order effect that is estimated on noisier withinperson deviations in activation and with less information than the main age trajectory. A basis dimension of k=10 smooth allows sufficient flexibility to capture moderate nonlinear variation in coupling across baseline age, while reducing the risk of overfitting. All other terms in the model were the same as in the activation model—notably the smooth of Time, *f*_2_, and tensor-product interaction smooth *f*_3_, which modeled longitudinal cognitive trajectories. This model was fitted separately for each brain region (354 memory ROIs; 356 fluid ROIs), with the same model specification applied to each region.

### 2.4 Statistical thresholding

For models with activation as predictor or outcome, we applied an uncorrected significance threshold of *p* = 0.005 to identify effects of interest. Our primary variable of interest across all models was the interaction term between Age × Time, either represented as the tensor-product smooth for activation and cognition-only models or as the smooth age-varying effect of activation in the cognition-activation models that included activation as a predictor. For cognition-only models, we report all variables and subscribe to an uncorrected threshold of *p* = 0.05. For activation and cognition–activation models, all terms are presented in tables only for models in which the interaction term was significant. Spline adequacy was evaluated using *gam.check*, focusing on whether each smooth had sufficient flexibility. We verified that the estimated effective edf did not reach the upper limit of the basis (k′) and that the *k*-index test was not significant. A significant *p*-value in this test indicates that the basis dimension may be too low and potential underfitting, whereas a non-significant result suggests that the chosen basis is adequate.

## 3. Results

Although the longitudinal GAMM was specified in mathematical form, all smooth terms in the Results tables and figures are labeled using mgcv/R notation for clarity. In this notation, s(Age_T0_) and s(Time) denote univariate smooth functions of baseline age and time, respectively; ti(Age_T0_, Time) denotes the tensor-product interaction smooth capturing their joint nonlinear effect; and s(Age_T0_, by = ROI_w_) denotes the smooth of baseline age that varies as a function of within-individual region activation (ROI_w_). We use this notation interchangeably with the mathematical expressions for ease of interpretation.

### 3.1 MEM

#### 3.1.1. Activation models

**Figure 2.**
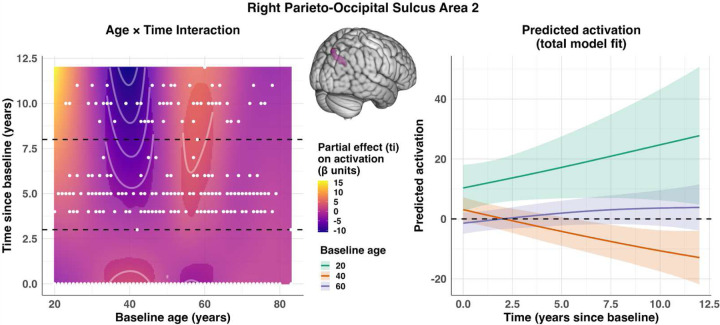
Interaction plot for the partial effect and predicted activation model fit for reference ages for the right Parieto-Occipital Sulcus Area 2. Left: The estimated partial effect of the Age × Time tensor-product smooth from the full GAMM (see Equation 1), plotted as a function of baseline age and time since baseline. Color indicates the contribution of the Age × Time interaction term to regional activation (in β units). Highlighted regions indicate regions where the Age × Time interaction term is statistically significant based on pointwise 95% confidence intervals. The contour lines are predominantly vertical and U-shaped, indicating that the interaction effect mainly reflects an age-related modulation of the time slope, with nonlinear peaks and valleys in the interaction surface that depend jointly on age and time. Dashed horizontal lines indicating the earliest measurement for each wave and marking the boundaries between wave-specific time windows. White points denote the observed distribution of baseline age and wave assessments, illustrating areas of strongest data support. Right: Predicted activation trajectories across follow-up time for three reference baseline ages (20, 40, 60 years), obtained from the full GAMM (including all smooths and fixed effects but excluding subject-specific random effects). For each reference age, activation was predicted across the observed time span while holding sex at its reference level and education at the sample mean. Curves illustrate how total model-estimated activation evolves over time for individuals of different baseline ages. Shaded bands represent pointwise 95% confidence intervals of the predicted activation trajectories, reflecting uncertainty in the estimated mean activation level at each time point.

We first present the significant findings from the activation models. A *p*= 0.005 uncorrected threshold yielded four regions where a significant Age × Time interaction ti(Age_T0_, Time) was found (see Table 2), with varying levels of nonlinear complexity. A relatively weak nonlinear interaction (*edf* = 1.892, *F* = 5.546, *p* = 0.003) was found in the left Intraparietal Sulcus Area 1, whereas the left Lateral Area 7A (*edf* = 3.116, *F* = 6.878, *p* < 0.001) and right Medial Area 7A (*edf* = 3.279, *F* = 4.425, *p* = 0.004) showed more pronounced nonlinearities. The right Parieto-Occipital Sulcus Area 2 (R POS2) displayed the strongest effect with a highly complex interaction surface (*edf* = 6.583, *F* = 3.279, *p* = 0.003), indicating substantial nonlinear variation in activation change across age and time. An illustration of the partial interaction effect and the model-predicted trajectories for three reference ages for the R POS2 is provided in Figure 2. The contour lines reflect how the interaction behaves: vertical contours indicate stronger age-related modulation of the time slope, horizontal contours indicate stronger time-related modulation of the age slope, diagonal contours reflect joint age–time influences, and U-shaped contours mark nonlinear peaks or valleys in the interaction surface (with their orientation showing whether curvature is primarily across age or across time). In this region, the significant contours were predominantly vertical and U-shaped, indicating that the interaction was driven mainly by age-related modulation of the time slope, with nonlinear peaks and valleys that depended jointly on age and time. Most notably, activation shows a sharp decline at later testing time points in midlife, a pattern that is further reflected in the longitudinal decreases observed when examining the GAMM-predicted trajectories by reference age. Contour plots for the three additional regions showing significant interactions are provided in the supplementary figure 1 (SF1).

#### 3.1.2 Cognition-only model

**Figure 3.**
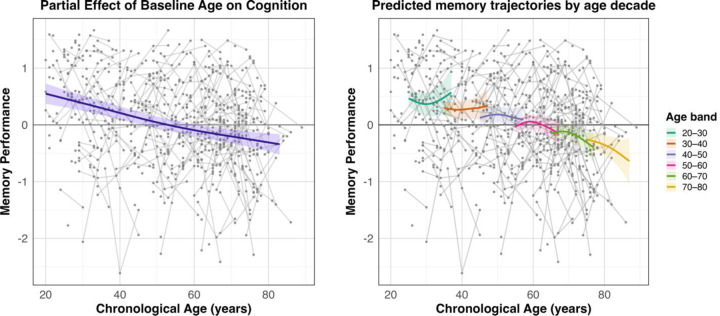
GAMM plots for the significant smooth age effect and predicted memory trajectories by decade. Left: Partial effect of baseline age on memory performance. The curve shows the estimated relationship between baseline age and memory at Time = 0, adjusted for sex and education. Shaded bands indicate 95% confidence intervals and gray lines show individual raw observations over chronological age (i.e., Age_T0_ + Time). Right: Predicted memory trajectories across chronological age for individuals entering the study at different baseline ages. Curves represent model-implied trajectories for each baseline-age decade, generated by fixing Age_T0_ to the midpoint of each decade, predicting memory across the observed time range, and expressing predictions in chronological age. These trajectories illustrate how predicted memory levels vary across baseline-age groups. Shaded areas indicate pointwise 95% confidence intervals.

Results for the cognition-only MEM model are reported in Table 3. There was a significant smooth effect of baseline age on memory performance (*p* < .001), indicating that memory performance declined with increasing age. Higher education was also associated with better performance (β = 0.090, *p* < .001). In contrast, neither the smooth effect of time (*P* = .359) nor the Age × Time interaction (*P* = .136) reached significance, suggesting that age did not reliably modify the longitudinal trajectory of memory performance. The partial effect of the age smooth, adjusted for all other model terms, is shown in Figure 3. Additionally, although the interaction was not significant, we plotted predicted memory trajectories stratified by age decade to illustrate model fit and natural variability in predicted memory levels across baseline-age groups without implying statistical differences in longitudinal slopes. These trajectories were generated from the full GAMM (excluding subject-specific random effects) by fixing baseline age to the midpoint of each decade and computing model-implied memory estimates across the observed time range.

#### 3.1.3 Cognition-activation models

**Figure 4.**
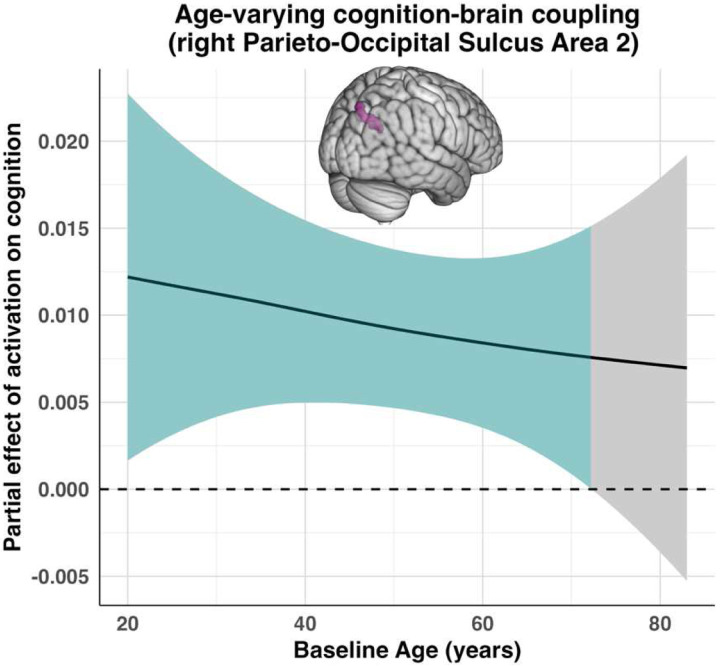
Age-varying coupling between activation and cognition in the right ParietoOccipital Sulcus Area 2 (R POS2). Plot displaying the estimated within-individual coupling between activation and cognition as a smooth function of baseline age, derived from the significant age-varying coefficient term in the cognition–activation model, for the right parieto-occipital sulcus area 2. Activation values reflect within-individual deviations from each participant’s own mean activation. The curve quantifies how the strength of the within-individual association between activation and cognition varies with baseline age. The shaded band represents the pointwise 95% confidence interval, with regions whose intervals exclude zero deemed significant. Positive values (green) indicate that, at a given age, increases in activation tend to be associated with improvements in cognition, whereas negative values indicate the opposite. Grey regions denote ages where the confidence interval includes zero, indicating that the coupling is not statistically significant.

Cognition models were re-estimated with each ROI’s activation entered as an additional predictor. Six regions emerged where the age-varying effect of activation on cognition was significant, as indexed by the varying-coefficient smooth (s(Age, by = ROI_w_)). A list of all smooth effects for the models displaying a significant age-varying activation–cognition coupling term are presented in Table 4. The strongest effect was observed in the right Parieto-Occipital Sulcus Area 2 (R POS2), a region that also emerged as displaying the most prominent nonlinear effect among the activation models tested. Here, the age-varying coupling was very robust (*edf* = 2.711, *F* = 6.076, *p* = 0.001), indicating that higher-than-expected activation predicted better cognition for a majority of the age span until around 70 years of age (see Figure 4), although the magnitude of this positive coupling steadily weakened with age. Area 23D, however, displayed a bilateral age-dependent modulation of the activation–cognition association, including a directional reversal in older adulthood. Both the left (*edf* = 2.608, *F* = 5.560, *p* = 0.003) and right (*edf* = 2.592, *F* = 5.055, *p* = 0.003) 23D regions showed significant positive coupling in young adulthood—more restricted to earlier ages in the left hemisphere—followed by a nonsignificant attenuation in midlife and a negative coupling in late adulthood, whereby higher-than-expected activation was associated with worse cognitive performance. Two other regions— the right Area 23C (*edf* = 2.502, *F* = 6.095, *p* = 0.003) and the right Area 31A (*edf* = 2.592, *F* = 5.055, *p* = 0.003)— displayed similar patterns to bilateral Area 23D. Finally, the left superior temporal visual area (L STV; *edf* = 2.71, *F* = 6.08, *p* = 0.001) displayed a significant negative cognitionactivation coupling in midlife (see supplementary figure 2; SF2).

### 3.2 FLUID

#### 3.2.1. Activation models

**Figure 6.**
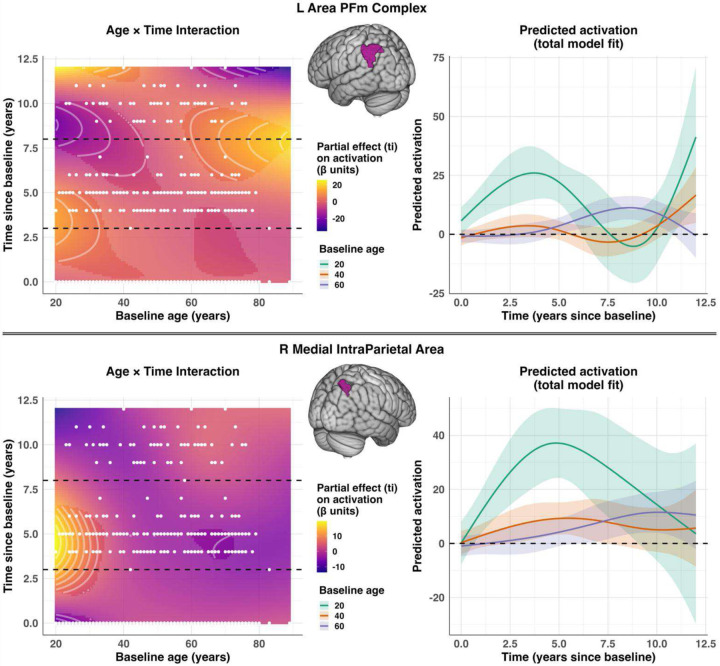
Interaction plot for the partial effect and predicted activation model fit for reference ages for two regions (upper = L Area PFm Complex (L PFm); lower=R Medial IntraParietal Area (R MIP)). Left plots: The estimated partial effect of the Age × Time tensor-product smooth from the full GAMM (see Equation 1), plotted as a function of baseline age and time since baseline. Color indicates the contribution of the Age × Time interaction term to regional activation (in β units). Highlighted regions indicate regions where the Age × Time interaction term is statistically significant based on pointwise 95% confidence intervals. For both the L PFm and R MIP, the contour lines are predominantly U-shaped, horizontally-oriented, indicating that the interaction effect varies more steeply across time than across age, reflecting stronger age-dependent modulation of the time slope. In the L PFm, younger adults exhibit early-follow-up increases in activation that diminish with increasing age, whereas older adults show activation increases primarily at later follow-up times. In the R MIP, a pronounced early-follow-up increase in activation is present only in younger adults, with minimal corresponding effects in middle-aged and older adults. Right plots: Predicted activation trajectories across follow-up time for three reference baseline ages (20, 40, 60 years), obtained from the full GAMM (including all smooths and fixed effects but excluding subject-specific random effects). For each reference age, activation was predicted across the observed time span while holding sex at its reference level and education at the sample mean. Curves illustrate how total model-estimated activation evolves over time for individuals of different baseline ages. Shaded bands represent pointwise 95% confidence intervals of the predicted activation trajectories. Plots highlight that the time-related activation slopes differ markedly by age, with the strongest nonlinear fluctuations occurring in younger adults.

Again, we first present the significant findings from the activation models. A *p*= 0.005 uncorrected threshold yielded thirty regions where a significant Age × Time interaction ti(Age_T0_, Time) was found, each with varying levels of nonlinear complexity. We report the full models for the regions displaying the higher nonlinear complexity (edf > 3; see Table 5). For plotting, we focus on the two regions with the highest nonlinear complexity and are most representative of the pattern of interaction results; however, a full panel of contour plots for regions where a significant interaction was evident can be found in supplementary figure 3 (SF3). A strong nonlinear interaction was found in the left Area PFm Complex (L PFm; *edf* = 6.436, *F* = 3.551, *p* = 0.002) and the right Medial IntraParietal Area (R MIP; *edf* = 5.460, *F* = 4.420, *p* = 0.001). However, more generally, all seven regions were confined to the higher order temporo-parietal association cortex, including dorsal parietal regions (L 7AL, L PFm, R MIP) and the auditory belt/parabelt complexes (L PBelt, L MBelt, L LBelt, R LBelt). An illustration of the partial interaction effect and the model-predicted trajectories for three reference ages for the L PFm and R MIP can be found in Figure 6. Both the L PFm and R MIP showed significant Age × Time interactions but with distinct temporal profiles. In the left PFm complex, although older adults displayed a localized positive interaction effect at mid-to-late follow-up, the largest overall changes in predicted activation occurred in younger adults, who displayed an initial increase followed by a marked decrease over the follow-up period. Estimates at the second follow-up should be interpreted cautiously, however, given the fewer observations at later time points. In contrast, the R MIP displayed a pronounced early-follow-up increase in activation that was present only in younger adults and diminished sharply with age, with little evidence for modulation at later follow-up years. However, it should be noted that most significant ROIs (see SF3), displayed a gradual age-dependent activation reversal— younger adults exhibited early decreases in activation, whereas older adults showed late increases, with middle-aged adults remaining relatively stable. This reflects a mild, age-dependent shift in activation trajectories rather than the sharply nonlinear patterns observed in the smaller subset of regions reported.

#### 3.1.2 Cognition-only model

**Figure 7.**
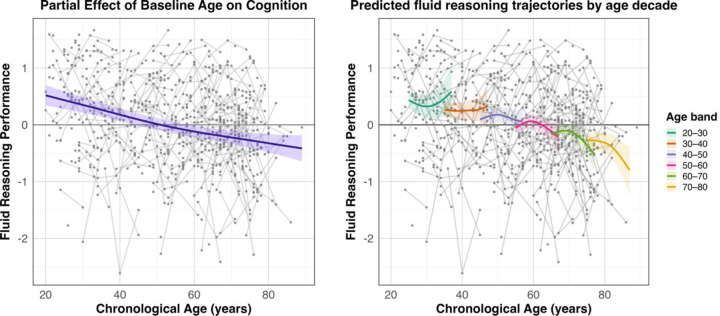
GAMM plots for the significant smooth age effect and predicted memory trajectories by decade. Left: Partial effect of baseline age on fluid reasoning performance. The curve shows the estimated relationship between baseline age and fluid reasoning at Time = 0, adjusted for sex and education. Shaded bands indicate 95% confidence intervals and gray lines show individual raw observations over chronological age (i.e., Age_T0_ + Time). Right: Predicted fluid reasoning trajectories across chronological age for individuals entering the study at different baseline ages. Curves represent model-implied trajectories for each baseline-age decade, generated by fixing Age_T0_ to the midpoint of each decade, predicting fluid reasoning across the observed time range, and expressing predictions in chronological age. These trajectories illustrate variability in predicted fluid reasoning across baseline-age groups, showing a general tendency toward age-related divergence over time despite the non-significant Age × Time interaction (P = .08). Shaded areas indicate pointwise 95% confidence intervals.

Results for the cognition-only FLUID model are reported in Table 3. There was a significant smooth effect of baseline age on fluid reasoning performance (*p* < .001), where higher baseline age was associated with worse performance. Conversely, higher education was associated with better performance (β = 0.090, *p* < .001). Neither the smooth effect of time (*P* = .167) nor the Age × Time interaction (*P* = .080) reached significance, although there was an evident trend for the latter. The partial effect of the age smooth, adjusted for all other model terms, is shown in Figure 7. Additionally, although the interaction was not significant, we again plotted predicted fluid reasoning trajectories stratified by age decade to illustrate the trend in variability in predicted fluid reasoning levels across baseline-age groups. These trajectories were generated from the full GAMM (excluding subject-specific random effects) by fixing baseline age to the midpoint of each decade and computing model-implied fluid reasoning estimates across the observed time range.

#### 3.1.3 Cognition-activation models

**Figure 8.**
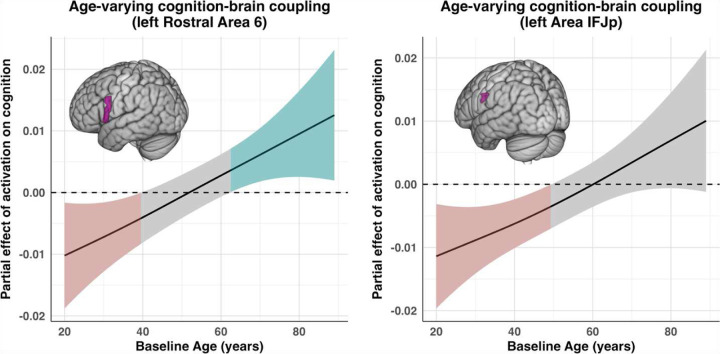
Age-varying cognition–brain coupling in left Rostral Area 6 and left Area IFJp. Plots display the estimated within-individual coupling between activation and cognition as a smooth function of baseline age. Activation values reflect within-individual deviations from each participant’s own mean activation. The curve represents the estimated partial effect of transient activation increases on corresponding fluctuations in cognition at each baseline age. Shaded regions denote pointwise 95% confidence intervals, with colors indicating ages at which the effect is significantly different from zero and gray regions denoting non-significant estimates. In the left Rostral Area 6 (left plot), activation is negatively associated with cognition in younger adults, but this relationship becomes progressively less negative with age and transitions into a significantly positive association in later adulthood. In left Area IFJp, the negative association observed in younger and early middle-aged adults attenuates with age but does not reach a significant positive effect in older age.

For cognition models re-estimated with each ROI’s activation entered as an additional predictor, four regions emerged as displaying a significant age-varying effect of activation on cognition (s(Age, by = ROI_w_)). A list of all smooth effects for these models are presented in Table 6. Interestingly, the strongest effect was observed in the right Parieto-Occipital Sulcus Area 2 (R POS2; *edf* = 2.377, *F* = 8.361, *p* < .001), a region that also appeared prominently in the MEM analyses, showing both nonlinear Age × Time effects on activation and age-varying cognition–brain coupling. As observed in the MEM domain, activation in R POS2 was positively associated with cognition in younger adults, with this relationship weakening and approaching zero or slightly negative values with increasing age. A similar age-varying pattern was observed in the right Parieto-Occipital Sulcus Area 1 (R POS1; *edf =* 2.311, *F* = 5.267, *p* = .003), and due to its conceptual similarity to the MEM findings, this pattern is reported descriptively rather than illustrated. In the left Rostral Area 6, the interaction term (*edf* = 2.312, *F* = 5.183, *p* = .004) indicated that activation was negatively related to cognition at younger ages but shifted toward a positive association in later life, yielding a monotonic age-related strengthening of the coupling (Figure 8, left). A similar pattern was observed in the left Area IFJp (*edf =* 2.299, *F* = 5.316, *p* = .003), where higher activation was significantly negatively associated with cognition in younger and early middle-aged adults; with increasing age, this negative relationship attenuated toward zero yet never reached a statistically significant positive association in later adulthood (see Figure 8).

## 4. Discussion

In the current study, we used GAMMs to characterize nonlinear trajectories of brain activation and cognition as well as to determine how moment-to-moment fluctuations in regional activation predict corresponding fluctuations in cognitive performance as a function of age. By leveraging the use of tensor-product smooths, our aim was to capture complex, nonlinear Age × Time patterns that would not be detectable using conventional linear or polynomial approaches. Together, our analyses revealed (1) distinctive nonlinear age-related changes in activation across high-order association cortices, (2) robust age effects on memory and fluid abilities, and (3) region-specific age-varying relationships between activation and cognition, with several regions showing non-linear directional changes, including sign reversals, across the adult lifespan.

Across both the MEM and FLUID domains, several higher-order association-cortical regions displayed significant nonlinear Age × Time interactions. Although the specific ROIs differed across domains, the interactions consistently localized to a set of regions in the dorsal parietal cortex, temporo-parietal belt, and parieto-occipital regions, all regions situated within the posterior association cortex. For the MEM domain, significant effects were observed in left IPS1, left 7AL, right POS2, and right 7Am, reflecting age-dependent modulation of longitudinal activation slopes with varying nonlinear complexity. Interestingly, Vidal-Piñeiro and colleagues (2020) identified several lifespan-continuous trajectories of memory encoding activity across the cortex, including both frontal and posterior association regions, but importantly showed that posterior heteromodal areas were among the most age-sensitive and exhibited pronounced developmental–aging continuity. Their main conclusion was that age differences in task activation largely reflect longstanding organizational constraints rather than late-life emergent mechanisms and did not find evidence for over-recruitment, compensation, nor negative activity-performance relationships. Similarly, our activation GAMMs showed Age × Time interactions confined to posterior association cortex and no evidence of frontal over-recruitment. However, in bilateral Area 23D—an area most closely aligned with the posterior cingulate cortex (PCC)— we observed an age-dependent reversal in cognition–activation coupling: in younger adults, within-person increases in activation were associated with better memory performance, whereas in older adults the same increases predicted poorer performance. Additionally, the left Area 23D also showed an age-related increase in activation (trend-level s(Age): *p* = 0.0052), indicating that older adults engage this region more strongly across the lifespan. Thus, although mean activation was lower in younger adults, increases in activation were associated with better performance, suggesting a shift from efficient, beneficial recruitment in youth to inefficient, maladaptive over-recruitment in later life. While this pattern does not reflect compensatory recruitment within the cognitive reserve framework (Stern, 2020; Stern, 2009), the combination of age-related upregulation and detrimental cognitive coupling is most consistent with maladaptive over-recruitment or failed compensation, in which greater activation reflects reduced neural efficiency or interference rather than support for performance (Cabeza et al., 2018; Morcom & Johnson, 2015). The PCC has been strongly implicated in task-related activation, showing consistent co-activation with prefrontal, parietal, and limbic regions during cognitive demands such as memory retrieval and attentional control (Busler et al., 2019; Leech & Sharp, 2014). In the context of aging, functional abnormalities in the PCC have also been observed during task performance (Prakash et al., 2012; Spreng and Schacter, 2012), with a failure of PCC *deactivation* that is coupled with cognitive load increases (Prakash et al., 2012). A similar pattern emerged in the left Superior Temporal Visual Area—extending from the superior temporal cortex into the medial temporal lobe—which showed both age-related increases in activation and a significant negative cognition–activation coupling specifically in midlife. This suggests that midlife may be a particularly sensitive period during which neural upregulation becomes decoupled from cognitive benefit. This interpretation is consistent with evidence that superior temporal and medial temporal regions support episodic memory (Eichenbaum et al., 2012) and show heightened age-related vulnerability in activation and connectivity (Cansino, 2022). Thus, the midlife-specific negative coupling may reflect an early disruption in temporal–MTL functional coordination, whereby increased activation no longer no longer confers cognitive benefit.

The right POS2—a region sharing overlap with the precuneus/cuneus— displayed the highest nonlinear trajectory, with activation reductions at later testing time points in midlife, suggesting that midlife may mark a period of functional reorganization in which the ability to sustain activation decreases disproportionately over time. The emergence of the right POS2 is noteworthy given the central role of the precuneus in episodic memory networks (Nyberg et al., 2017; Wagner et al., 2005) and its well-documented susceptibility to age-related structural and functional decline—including reductions in retrieval-related activation and alterations in largescale network integration—in both healthy aging (Wang et al., 2010) and individuals along the Alzheimer’s disease spectrum (Celone et al., 2006). The right POS2 also displayed a significant age-related smooth, reflecting a monotonic decline in activation from young adulthood into old age that is consistent with normative posterior aging rather than compensatory over-recruitment (Reuter-Lorenz & Park, 2010; Reuter-Lorenz & Cappell, 2008; Cabeza et al., 2018). The nonlinear midlife downturn we observed in the right POS2 may therefore reflect age-dependent changes in a region that plays a pivotal role in memory-related posterior cortical processes and is consistent with the literature identifying the precuneus as a sensitive locus of age-related vulnerability in episodic memory.

Within the FLUID domain, two posterior parietal regions—the left PFm complex and the right medial intraparietal area (MIP)—showed pronounced age × time interactions in activation. This localization is highly consistent with the parieto-frontal integration theory of intelligence, which posits that inferior and superior parietal cortex and precuneus form core posterior hubs of the fronto-parietal system supporting fluid reasoning (Santarnecchi et al., 2017; Vakhtin et al., 2014). Meta-analytic and network-level work has demonstrated that these posterior parietal regions are reliably recruited by demanding reasoning tasks and that their integrity tracks individual differences in fluid intelligence. Both regions showed pronounced activation increases early in younger adults, consistent with efficient recruitment of parieto-frontal circuitry during periods of maximal fluid reasoning capacity. However, activation trajectories differed later in adulthood: the left PFm displayed attenuated fluctuations in midlife but later-life increases whereas the right MIP displayed large early-follow-up increase exclusively in young adults, with this effect diminishing with advancing age. Our findings suggest that, although the left PFm and right MIP are critically involved in fluid reasoning, their engagement becomes less efficient and more heterogeneous with age, possibly attesting to age-related reductions in specificity or neural reorganization rather than a simple monotonic decline.

We additionally identified regions showing significant age-varying cognition–activation coupling in the left IFJp and left rostral Area 6, where the direction of coupling shifted from negative in younger adults to positive in later adulthood. Although these regions remained functionally relevant across the lifespan, with older adults exhibiting greater cognitive benefit from activation increases, this pattern does not align with neural reserve. Neural reserve presupposes stable efficiency and a consistent positive relationship between activation and performance across age (Stern, 2009). In contrast, the observed reversal suggests that recruitment of this network is inefficient or even maladaptive in younger adults but becomes advantageous only in later adulthood. This interpretation is further supported by a significant age-related decline in IFJp activation, demonstrating that the region does not maintain stable activation levels over time. This age-contingent shift is more consistent with capacity-limited compensatory recruitment, whereby older adults increasingly rely on alternative circuitry as primary systems weaken, rendering upregulation of IFJp beneficial only under conditions such as reduced neural resources (Reuter-Lorenz & Park, 2014). Notably, the right POS2 (precuneus/cuneus) also emerged as a region showing significant cognition–activation coupling in the FLUID domain, displaying a pattern identical to that observe do the MEM domain. The recurrence of the right POS2 across both domains highlights its broad functional role not only as fundamental to episodic retrieval (Nyberg et al., 2017) but as a domain-general hub involved in attentional allocation, integration of internal and external information, and transitions between default mode and task-positive states (Spreng et al., 2013). Its engagement across both memory and fluid reasoning is consistent with evidence that it functions as a domain-general hub supporting attentional allocation and task control, rather than serving a process-specific role.

Beyond POS2, several additional regions exhibited significant Age × Time tensor-product interactions in the FLUID domain. This more extensive set of interaction effects likely reflects the distributed nature of the neural architecture supporting fluid reasoning, which relies on coordinated engagement of posterior parietal, temporo-parietal, and frontal systems. Prior work has consistently shown that fluid reasoning is subserved by a broad parieto-frontal network (Colom et al., 2010; Santarnecchi et al., 2017; Vakhtin et al., 2014), and the heterogeneous pattern of nonlinear age-dependent trajectories observed here aligns with the view that fluid reasoning relies on multiple interacting cortical subsystems that may exhibit distinct age-related dynamics and differential susceptibility to neural reorganization across adulthood.

In addition to the longitudinal interaction effects, we also examined cross-sectional age differences as indexed by the smooth term s(Age_T0_). These effects revealed a broad pattern of age-related decreases in frontal regions and age-related increases in posterior regions (see Supplementary Figure 4; SF4). However, the longitudinal Age × Time interactions presented a far more selective pattern: significant nonlinear age-related modulation of activation over time was confined almost exclusively to posterior association cortex in both MEM and FLUID domains, including dorsal parietal, PFm/belt, and parieto-occipital regions, with no evidence of dynamic age-related modulation in frontal cortex. Thus, although cross-sectional age differences appear widespread, the regions showing longitudinal age-dependent change were more circumscribed and consistently posterior. This posterior selectivity converges with prior work from our lab, where age-weighted longitudinal maps similarly revealed little evidence for frontal age-related increases. Instead, longitudinal activation increases—and their age sensitivity—were predominantly localized to posterior association regions, particularly in later adulthood (Argiris et al., 2021). In the present work, we extend this finding by demonstrating that specific posterior regions exhibit nonlinear, age-dependent longitudinal dynamics, offering a more precise characterization of when along the adult lifespan these changes occur. Furthermore, by modeling age-varying cognition–activation coupling, we directly linked these activation changes to corresponding cognitive gains or declines, providing functional interpretability for the observed neural dynamics. Taken together, our findings contrast with the posterior–anterior shift in aging (PASA) model (Davis et al., 2008) and with compensatory interpretations emphasizing increased prefrontal recruitment in older adults (e.g., Cabeza et al., 1997, 2002; Reuter-Lorenz et al., 2000; Grady et al., 2005), as both domains suggest that age-related changes in activation— particularly those characterized by nonlinear patterns of change over time—largely reflect lifespan trajectories of posterior association systems rather than compensatory increases in engagement of frontal regions.

When examining the cognition-only models, smooth effects of baseline age were observed for both MEM and FLUID, with performance showing a largely monotonic decline across the lifespan. Although the Age × Time interaction was not statistically significant in either domain, plots of predicted trajectories stratified by baseline-age decade revealed meaningful variability in both MEM and FLUID levels, with FLUID demonstrating the most pronounced age-related pattern (trend-level, P = 0.08). his overall monotonic pattern is compatible with longitudinal work showing that early and mid-adulthood often exhibit gradual decline, with clearer nonlinear acceleration typically emerging later in life or over longer follow-up intervals (LaPlume et al., 2022; Salthouse, 2019; Lindenberger, 2014). The subtle decade-level differences observed here may therefore reflect early hints of this transition, suggesting that nonlinearities in cognitive change are present but modest and not statistically reliable relative to the more pronounced age-dependent inflections observed in activation trajectories. This pattern may also reflect the comparatively limited number of participants who completed all three testing waves, which likely reduces sensitivity to detecting nonlinear cognitive change over time.

A critical future direction will be to incorporate measures of structural integrity into GAMM-based analyses. Accounting for age-related gray-matter changes—such as cortical thinning, volumetric loss, or white-matter deterioration—will be essential for determining whether the age-dependent functional patterns observed here reflect compensatory recruitment, declining neural efficiency, or emerging neuropathology. Moreover, any evaluation of cognitive reserve (CR) mechanisms must situate functional–cognitive relationships within the broader context of structural brain integrity. Incorporating structural and lifestyle indicators into future longitudinal models will therefore be crucial for identifying whether and how reserve processes shape functional trajectories across adulthood. Given the emerging observations that midlife may mark a period of heightened functional reorganization or instability across multiple regions, greater emphasis should be placed on midlife transitions to identify early markers of maladaptive or compensatory neural change. Future work should examine how midlife experiences shape subsequent functional brain trajectories. Longitudinal population studies increasingly highlight midlife as a critical window for determining later-life cognitive outcomes, with lifestyle factors such as physical activity, social engagement, and cognitively stimulating behaviors predicting slower decline in episodic memory and executive abilities (e.g., Wang, Zhou, & Li, 2023). Consistent with this view, recent large-scale neuroimaging work indicates that brain aging itself follows nonlinear transitions beginning in midlife, suggesting a neural “critical window” for intervention (Antal et al., 2025). Incorporating comparable lifestyle measures into GAMM-based functional analyses will be essential for testing whether such factors modify the baseline level and the rate of change in activation trajectories, or whether they moderate age-varying cognition–activation coupling. Finally, future studies should incorporate a full bivariate varyingcoefficient term, s(Age_T0_, Time, by = ROI_w_), once larger samples with complete three-wave data become available. Although the s(Age_T0_, by = ROI_w_) term captured nonlinear age-varying coupling, the current dataset—containing only three measurement occasions with uneven follow-up—did not provide enough observations to reliably estimate how activation–cognition coupling might vary simultaneously across age *and* time (e.g., whether the coupling differs across follow-up waves).

## Supplementary Material

1

## Figures and Tables

**Figure 5.**
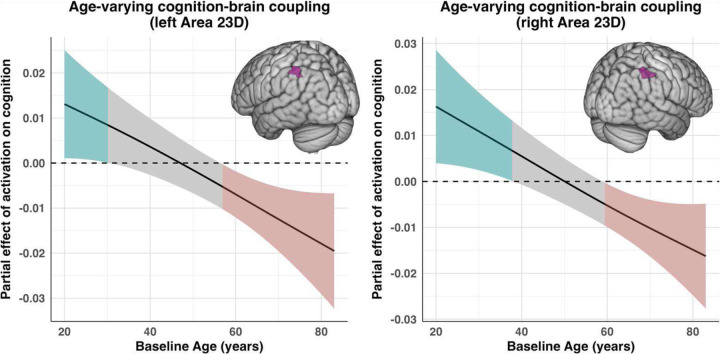
Age-varying cognition–brain coupling in bilateral Area 23D. Plots display the estimated within-individual coupling between activation and cognition as a smooth function of baseline age, derived from the significant age-varying coefficient term s(Age, by = ROI_w_) in the cognition–activation models, for the left and right Area 23D. Activation values reflect within-individual deviations from each participant’s own mean activation. The curve represents the estimated partial effect of within-individual fluctuations in activation on corresponding fluctuations in cognition at each baseline age. Shaded regions indicate pointwise 95% confidence intervals, with colors denoting whether the effect is significantly different from zero. Both hemispheres show a similar pattern: positive values (green) in young adulthood indicate that increases in activation are associated with improvements in cognition; a non-significant gray band in midlife reflects an attenuation of this association; and negative values (red) in older adulthood indicate that increases in activation are associated with worse cognition. Gray regions denote ages where the confidence interval includes zero, indicating non-significant coupling.

**Table 1.** Participant demographics. *Upper panel:* Table of availability of longitudinal data across the three study waves (T0–T2) and the corresponding number of participants contributing Memory and Fluid Reasoning data at each pattern of assessment completion. A star (✦) indicates that the participant contributed data at that time point. *Lower panel:* Table of baseline demographic and cognitive characteristics for participants grouped into three age brackets (i.e., 20–40 years, 40–60 years, and 61+ years) with corresponding counts (N, n). Baseline values were defined as each individual’s earliest available assessment, irrespective of the number of time point contributions. Mean (M) and standard deviation (SD) are reported for education (Edu) and cognition (Cog).

TO	T1	T2	Mem (N)	Fluid (N)
✦			167	175
	✦		36	30
		✦	9	8
✦	✦		129	137
✦		✦	13	14
	✦	✦	26	23
✦	✦	✦	51	54

Domain	Age Brackets (years)	N	Sex [n (% female)]	Edu [M (SD)]	Cog [M (SD)]
Memory	20–40	127	81 (63.78)	15.47 (2.39)	0.39 (0.77)
	40–60	135	71 (52.59)	15.67 (2.26)	0.01 (0.70)
	61+	170	89 (52.35)	16.20 (2.46)	−0.14 (0.69)
Fluid	20–40	135	88 (65.19)	15.47 (2.33)	0.35 (0.78)
	40–60	132	71 (53.79)	15.72 (2.26)	0.01 (0.68)
	61+	172	91 (52.91)	16.16 (2.46)	−0.16 (0.68)

**Table 2.** GAMM results for four Glasser atlas regions (L IPS1, L 7AL, R POS2, and R 7Am) for the MEM domain. Parametric estimates (i.e., Intercept, Sex, Edu) are presented with their associated standard errors, t-values, and p-values. Smooth terms for Time, Age_T0_, and their tensor interaction (ti(Age_T0_, Time)) are reported with F-statistics, estimated degrees of freedom (edf), and corresponding p-values.

ROI	Effect type	Term	Estimate	SE	df / edf	t / F	p-value
**L IntraParietal Sulcus Area 1 (L IPS1)**	Parametric	Intercept	−8.246	6.142	1.000	−1.342	0.180
		Sex	−0.063	1.850	1.000	−0.034	0.973
		Edu	0.400	0.379	1.000	1.055	0.292
	Smooth	s(Time)	—	—	1.734	1.479	0.138
		s(Age_T0_)	—	—	1.010	3.648	0.056
		**ti(Age**_**T0**_ **, Time)**	**—**	**—**	**1.892**	**5.546**	**0.003**
**L Lateral Area 7A (L 7AL)**	Parametric	Intercept	−1.192	4.884	1.000	−0.244	0.807
		Sex	−0.083	1.460	1.000	−0.057	0.955
		Edu	−0.037	0.301	1.000	−0.123	0.902
	Smooth	s(Time)	—	—	1.001	11.063	0.001
		s(Age_T0_)	—	—	1.027	5.349	0.019
		**ti(Age**_**T0**_ **, Time)**	**—**	**—**	**3.116**	**6.878**	**0.000**
**R Parieto-Occipital Sulcus Area 2 (R POS2)**	Parametric	Intercept	−2.975	4.996	1.000	−0.596	0.552
		Sex	−3.645	1.508	1.000	−2.417	0.016
		Edu	0.260	0.308	1.000	0.846	0.398
	Smooth	s(Time)	—	—	1.121	0.703	0.366
		s(Age_T0_)	—	—	4.061	4.211	0.003
		**ti(Age**_**T0**_ **, Time)**	**—**	**—**	**6.583**	**3.279**	**0.003**
**R Medial Area 7A (R 7Am)**	Parametric	Intercept	−7.284	4.796	1.000	−1.519	0.129
		Sex	0.505	1.440	1.000	0.351	0.726
		Edu	0.348	0.296	1.000	1.178	0.239
	Smooth	s(Time)	—	—	1.002	5.286	0.022
		s(Age_T0_)	—	—	1.011	6.023	0.014
		**ti(Age**_**T0**_ **, Time)**	**—**	**—**	**3.279**	**4.425**	**0.004**

**Table 3.** GAMM results for the cognition-only models (Memory and Fluid Reasoning). Parametric estimates (i.e., Intercept, Sex, Edu) are presented with their associated standard errors, t-values, and p-values. Smooth terms for Time, Age_T0_, and their tensor interaction (ti(Age_T0_, Time)) are reported with F-statistics, estimated degrees of freedom (edf), and corresponding p-values.

Domain	Effect type	Term	Estimate	SE	df / edf	t / F	p-value
**Memory**	Parametric	Intercept	−1.399	0.212	1.000	−6.606	0.000
		Sex	0.037	0.065	1.000	0.575	0.565
		Edu	0.090	0.013	1.000	6.934	0.000
	Smooth	s(Time)	—	—	1.585	0.601	0.359
		s(Age_T0_)	—	—	1.006	58.421	0.000
		**ti(Age**_**T0**_ **, Time)**	—	—	**3.359**	**1.864**	**0.136**
**Fluid Reasoning**	Parametric	Intercept	−1.405	0.210	1.000	−6.705	0.000
		Sex	0.051	0.064	1.000	0.802	0.423
		Edu	0.090	0.013	1.000	6.948	0.000
	Smooth t	s(Time)	—	—	1.837	1.420	0.167
		s(Age_T0_)	—	—	1.003	55.957	0.000
		**ti(Age**_**T0**_ **, Time)**	—	—	**3.608**	**2.091**	**0.080**

**Table 4.** GAMM results of the smooth terms only in the cognition-activation models with MEM as the outcome. Smooth terms for Time, Age_T0_, the tensor interaction (ti(Age_T0_, Time)), and s(Age_T0_, by=ROI_w_) are reported with F-statistics, estimated degrees of freedom (edf), and corresponding p-values.

ROI	Term	edf	F	p-value
**L Superior Temporal Visual Area (L STV)**	s(Time)	1.593	0.480	0.428
	s(Age_T0_)	1.006	58.821	0.000
	ti(Age_T0_, Time)	3.546	1.997	0.106
	**s(Age**_**T0**_ **, by=ROI**_**w**_ **)**	**2.711**	**6.076**	**0.001**
**L Area 23D (L 23D)**	s(Time)	1.655	0.951	0.240
	s(Age_T0_)	1.006	56.894	0.000
	ti(Age_T0_, Time)	3.997	2.607	0.035
	**s(Age**_**T0**_ **, by=ROI**_**w**_ **)**	**2.608**	**5.560**	**0.003**
**R Parieto-Occipital Sulcus Area 2 (R POS2)**	s(Time)	1.328	0.180	0.639
	s(Age_T0_)	1.006	53.000	0.000
	ti(Age_T0_, Time)	3.544	2.480	0.055
	**s(Age**_**T0**_ **, by=ROI**_**w**_ **)**	**2.645**	**8.194**	**0.000**
**R Area 23D (L 23D)**	s(Time)	1.662	1.070	0.210
	s(Age_T0_)	1.006	58.602	0.000
	ti(Age_T0_, Time)	4.027	2.411	0.047
	**s(Age**_**T0**_ **, by=ROI**_**w**_ **)**	**2.637**	**5.526**	**0.002**
**R Area 23C (R 23C)**	s(Time)	1.543	0.689	0.319
	s(Age_T0_)	1.006	55.984	0.000
	ti(Age_T0_, Time)	3.819	2.127	0.085
	**s(Age**_**T0**_ **, by=ROI**_**w**_ **)**	**2.502**	**6.095**	**0.003**
**R Area 31A (31A)**	s(Time)	1.494	0.524	0.391
	s(Age_T0_)	1.006	58.603	0.000
	ti(Age_T0_, Time)	3.636	2.257	0.079
	**s(Age**_**T0**_ **, by=ROI**_**w**_ **)**	**2.592**	**5.055**	**0.003**

**Table 5.** GAMM results for seven Glasser atlas regions (L 7AL, L PBelt, L PFm, L MBelt, L LBelt, R MIP, and R LBelt) for the FLUID domain. Parametric estimates (i.e., Intercept, Sex, Edu) are presented with their associated standard errors, t-values, and p-values. Smooth terms for Time, Age_T0_, and their tensor interaction (ti(Age_T0_, Time)) are reported with F-statistics, estimated degrees of freedom (edf), and corresponding p-values.

ROI	Effect type	Term	Estimate	SE	df / edf	t / F	p-value
**L Lateral Area 7A (L 7AL)**	Parametric	Intercept	2.405	4.517	1.000	0.533	0.595
		Sex	0.677	1.344	1.000	0.504	0.614
		Edu	−0.016	0.278	1.000	−0.056	0.955
	Smooth	s(Time)	—	—	1.001	44.088	0.000
		s(Age_T0_)	—	—	1.007	10.194	0.001
		**ti(Age**_**T0**_ **, Time)**	**—**	**—**	**4.650**	**4.160**	**0.001**
**L Parabelt Complex (L PBelt)**	Parametric	Intercept	−2.860	3.346	1.000	−0.855	0.393
		Sex	−1.759	0.993	1.000	−1.772	0.077
		Edu	0.079	0.206	1.000	0.383	0.702
	Smooth	s(Time)	—	—	1.760	31.588	0.000
		s(Age_T0_)	—	—	1.504	5.222	0.030
		**ti(Age**_**T0**_ **, Time)**	**—**	**—**	**3.145**	**5.975**	**0.000**
**L Area PFm Complex (L PFm)**	Parametric	Intercept	6.979	4.202	1.000	1.661	0.097
		Sex	−1.499	1.250	1.000	−1.200	0.231
		Edu	−0.302	0.259	1.000	−1.167	0.244
	Smooth	s(Time)	—	—	1.872	18.827	0.000
		s(Age_T0_)	—	—	3.300	4.656	0.003
		**ti(Age**_**T0**_ **, Time)**	**—**	**—**	**6.436**	**3.551**	**0.002**
**L Medial Belt Complex (L MBelt)**	Parametric	Intercept	−5.403	4.115	1.000	−1.313	0.190
		Sex	−1.663	1.222	1.000	−1.360	0.174
		Edu	0.377	0.253	1.000	1.488	0.137
	Smooth	s(Time)	—	—	1.002	0.207	0.651
		s(Age_T0_)	—	—	1.008	3.325	0.068
		**ti(Age**_**T0**_ **, Time)**	**—**	**—**	**3.937**	**4.384**	**0.002**
**L Lateral Belt Complex (L LBelt)**	Parametric	Intercept	1.715	3.380	1.000	0.507	0.612
		Sex	−2.344	1.004	1.000	−2.335	0.020
		Edu	−0.211	0.208	1.000	−1.016	0.310
	Smooth	s(Time)	—	—	1.741	41.881	0.000
		s(Age_T0_)	—	—	1.008	3.660	0.056
		**ti(Age**_**T0**_ **, Time)**	**—**	**—**	**3.773**	**4.357**	**0.004**
**R Medial IntraParietal Area (R MIP)**	Parametric	Intercept	9.912	6.349	1.000	1.561	0.119
		Sex	−2.384	1.889	1.000	−1.262	0.207
		Edu	−0.403	0.391	1.000	−1.030	0.303
	Smooth	s(Time)	—	—	1.980	16.905	0.000
		s(Age_T0_)	—	—	2.306	4.075	0.021
		**ti(Age**_**T0**_ **, Time)**	**—**	**—**	**5.460**	**4.420**	**0.001**
**R Lateral Belt Complex (R LBelt)**	Parametric	Intercept	−4.350	3.866	1.000	−1.125	0.261
		Sex	0.349	1.146	1.000	0.304	0.761
		Edu	0.314	0.238	1.000	1.319	0.187
	Smooth	s(Time)	—	—	1.000	6.092	0.014
		s(Age_T0_)	—	—	1.009	0.744	0.391
		**ti(Age**_**T0**_ **, Time)**	**—**	**—**	**3.201**	**5.304**	**0.001**

**Table 6.** GAMM results of the smooth terms only in the cognition-activation models with FLUID as the outcome. Smooth terms for Time, Age_T0_, the tensor interaction (ti(Age_T0_, Time)), and s(Age_T0_, by=ROI_w_) are reported with F-statistics, estimated degrees of freedom (edf), and corresponding p-values.

ROI	Term	edf	F	p-value
**L Rostral Area 6 (R 6R)**	s(Time)	1.003	55.947	0.000
	s(Age_T0_)	1.841	1.450	0.163
	ti(Age_T0_, Time)	3.504	2.407	0.053
	**s(Age**_**T0**_ **, by=ROI**_**w**_ **)**	**2.312**	**5.183**	**0.004**
**L Area IFJp (L IFJp)**	s(Time)	1.003	58.738	0.000
	s(Age_T0_)	1.940	2.311	0.089
	ti(Age_T0_, Time)	3.472	2.194	0.074
	**s(Age**_**T0**_ **, by=ROI**_**w**_ **)**	**2.299**	**5.316**	**0.003**
**R Parieto-Occipital Sulcus Area 2 (R POS2)**	s(Time)	1.003	47.718	0.000
	s(Age_T0_)	1.549	6.673	0.002
	ti(Age_T0_, Time)	3.927	3.159	0.015
	**s(Age**_**T0**_ **, by=ROI**_**w**_ **)**	**2.377**	**8.361**	**0.000**
**R Parieto-Occipital Sulcus Area 1 (R POS1)**	s(Time)	1.003	52.279	0.000
	s(Age_T0_)	1.704	3.899	0.014
	ti(Age_T0_, Time)	3.630	2.460	0.049
	**s(Age**_**T0**_ **, by=ROI**_**w**_ **)**	**2.311**	**5.267**	**0.003**

## Data Availability

The data that support the findings of this study are available from the corresponding author upon reasonable request. Custom-written code detailing analysis can also be made available upon reasonable request.
