# Supplementary material for: Nonlinear Lifespan Trajectories of Memory and Fluid Reasoning: A Longitudinal GAMM Study
Source: Res Sq. 2025 Dec 11:rs.3.rs-8313556. Preprint. [Version 1] doi: 10.21203/rs.3.rs-8313556/v1 (PMC12776469; doi:10.21203/rs.3.rs-8313556/v1)
Supplement: 1 [file NIHPPRS8313556V1-supplement-1.pdf]

Supplementary Material

Parameter	GE	Philips	Siemens
**TR (s)**	1	2	1
**TE (s)**	0.03	0.02	0.037
**Flip angle (°)**	72	72	52
**FOV (mm)**	220	240	208
**Matrix size**	110 × 110	112 × 112	104 × 104
**Voxel size (mm^3)**	2 × 2 × 2	2 × 2 × 4	2 × 2 × 2
**Number of slices**	64	33	72
**Pixel bandwidth**	4545.45	2447.38	2290
**Multiband / Acceleration factor**	—	—	6

ST1. Acquisition parameters for the three MRI scanners used across study waves (GE, Philips, Siemens). Table reports the repetition time (TR) and echo time (TE), in seconds (s), flip angle, field of view (FOV), in-plane matrix size, voxel dimensions, number of slices, pixel bandwidth, and multiband/acceleration factors for each scanner used across testing waves.

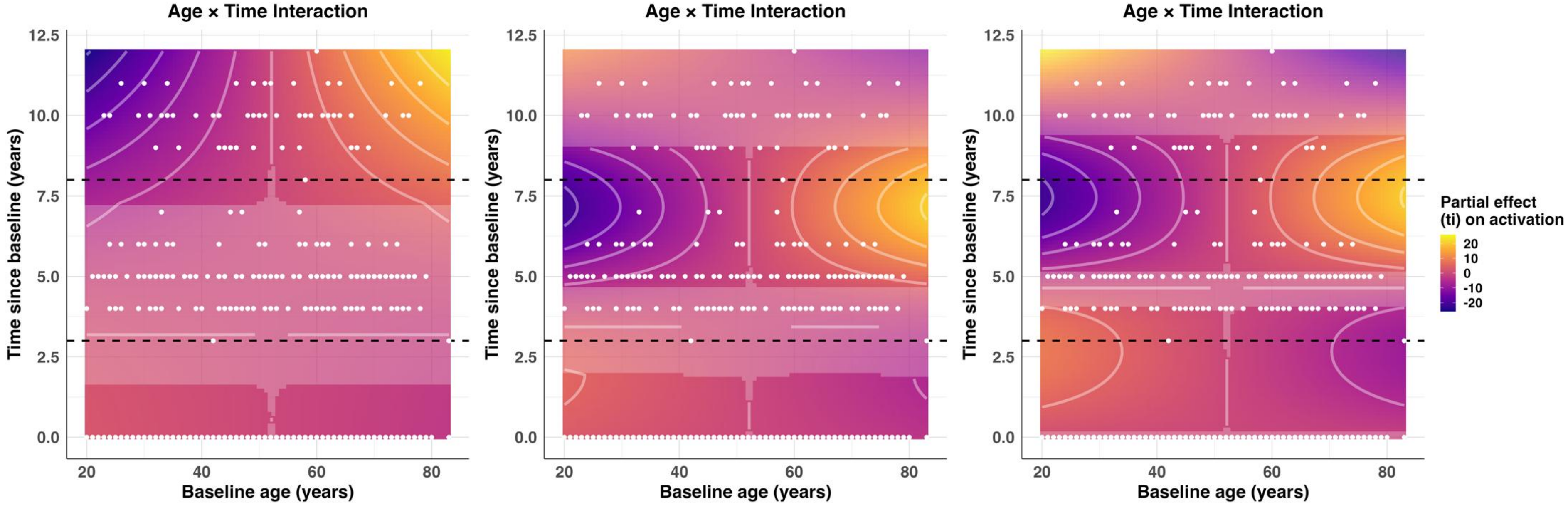

SF1. Partial effect surface of the Age × Time interaction on regional activation in the left IntraParietal Sulcus Area 1 (plot 1), left lateral area 7A (plot 2) and right Medial Area 7A (plot 3). *The* surface displays the estimated partial contribution of the Age × Time interaction from the fitted GAMM, obtained while holding all other fixed effects at their reference values (Sex = male; Education = sample mean) and using population-level estimates (i.e., random effects set to zero). Legend values represent the partial effect of the interaction on activation, expressed in β units, with color representing the magnitude and direction of that effect. Significance was assessed pointwise, and locations where the 95% confidence interval for the interaction excluded zero are displayed at full opacity, with non-significant areas shown more transparently.

White contour lines illustrate the structure of the interaction surface: U-shaped patterns mark local peaks or valleys; more vertical contours reflect stronger modulation by time; more horizontal contours reflect stronger modulation by age; and diagonal contours indicate that both variables jointly shape the interaction. White dots show the observed combinations of baseline age and time for all participants.

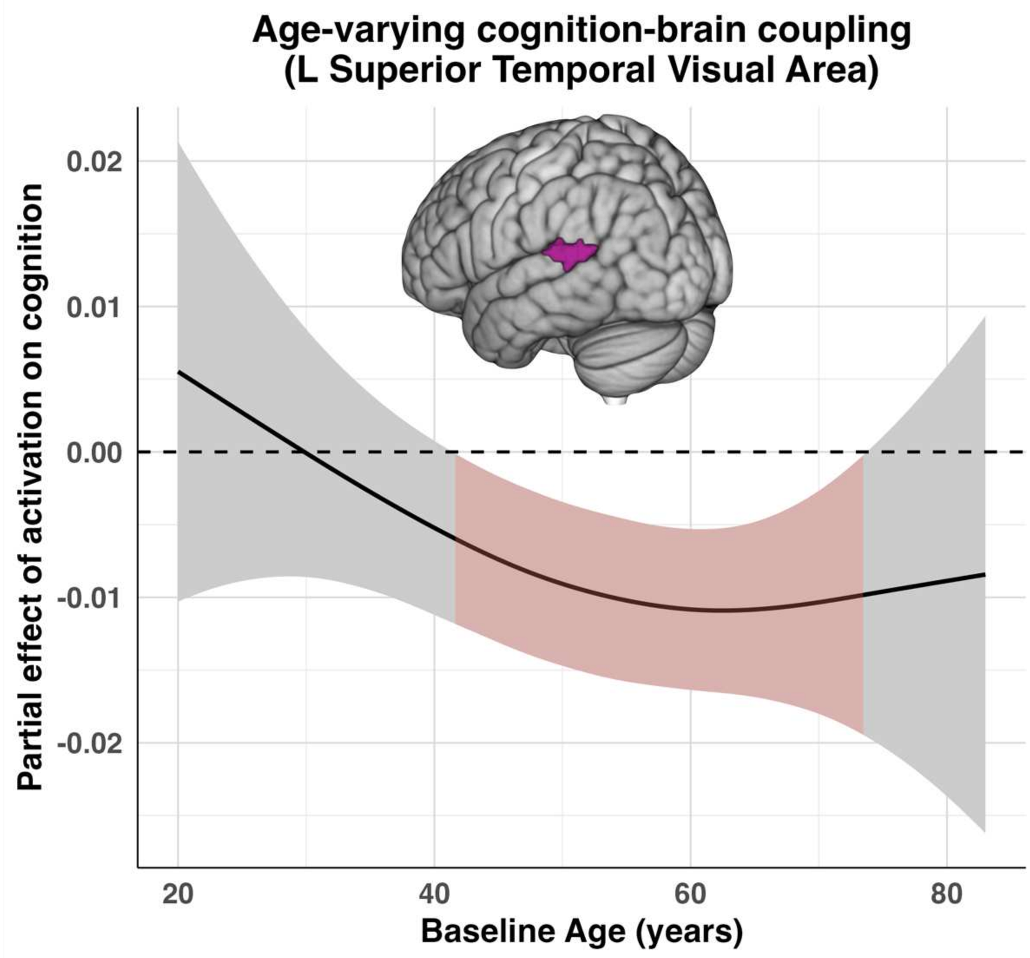

SF2. Age-varying cognition–brain coupling in the left Superior Temporal Visual Area for the MEM domain.

The plot shows the estimated within-individual coupling between activation and cognition as a smooth function of baseline age, derived from the significant age-varying coefficient term in the cognition–activation model for the left Superior Temporal Visual Area. Activation values reflect within-individual deviations from each participant's own mean activation. The curve represents the partial effect of these activation fluctuations on corresponding fluctuations in cognition at each baseline age. Shaded regions indicate pointwise 95% confidence intervals, with color denoting whether the effect differs significantly from zero. Notably, there is a significant negative coupling in midlife (red), indicating that increases in activation are associated with poorer cognitive performance. Gray regions denote ages where the confidence interval includes zero, indicating non-significant coupling.

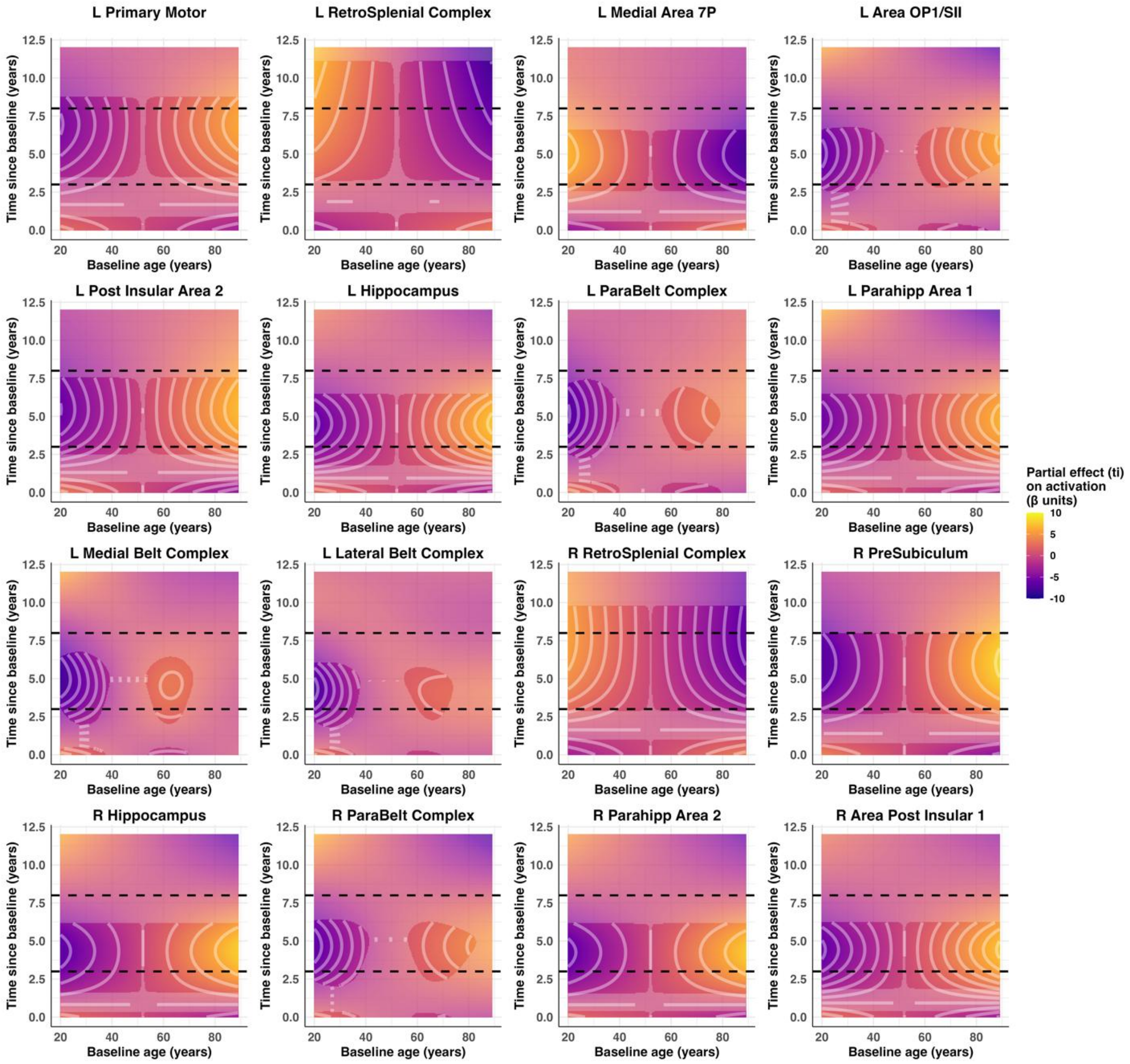

L Primary Motor
L RetroSplenial Complex
L Medial Area 7P
L Area OP1/SII
L Post Insular Area 2
L Hippocampus
L ParaBelt Complex
L Parahipp Area 1
L Medial Belt Complex
L Lateral Belt Complex
R RetroSplenial Complex
R PreSubiculum
R Hippocampus
R ParaBelt Complex
R Parahipp Area 2
R Area Post Insular 1
Time since baseline (years)
Baseline age (years)
Partial effect (ti) on activation (β units)
10
5
0
-5
-10

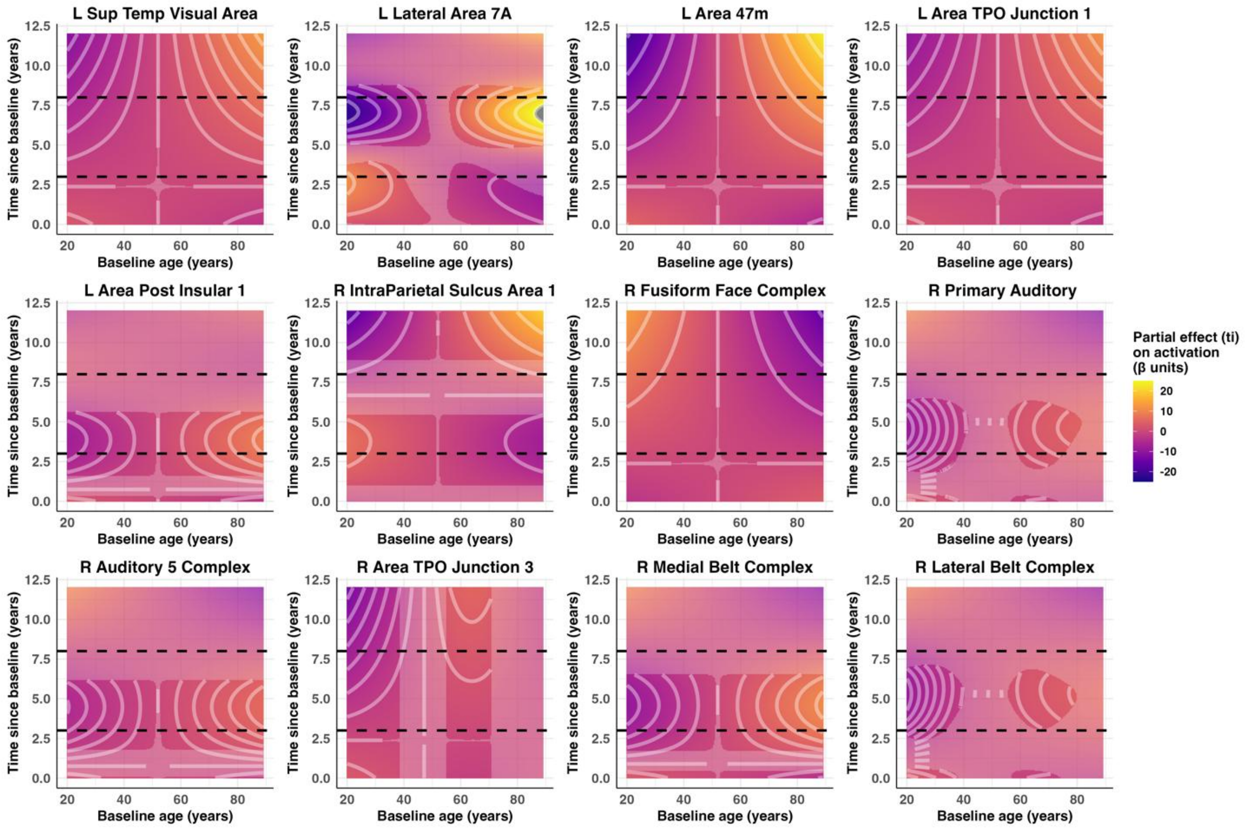

SF3. Panels of significant tensor-product smooth (ti(Age_{T0}, Time)) for 28 significant ROIs for activation models from the FLUID analysis (3.2.1) . The estimated partial effect of the Age × Time tensor-product smooth from the full GAMM, plotted as a function of baseline age and time since baseline. Color indicates the contribution of the Age × Time interaction term to regional activation (in β units). Areas where the 95% pointwise confidence interval does not include zero are shown at full opacity; more transparent areas indicate non-significant regions of the surface. Horizontal dashed lines denote the earliest observed times at which second- and third-visit assessments occur in the sample. Two separate color scales were used to accommodate differences in the range of tensor-product effects across regions. Across ROIs, a common pattern emerged— younger adults showed early follow-up decreases in activation, whereas older adults tended to show increases later in follow-up, with middle-aged adults exhibiting comparatively little longitudinal change.

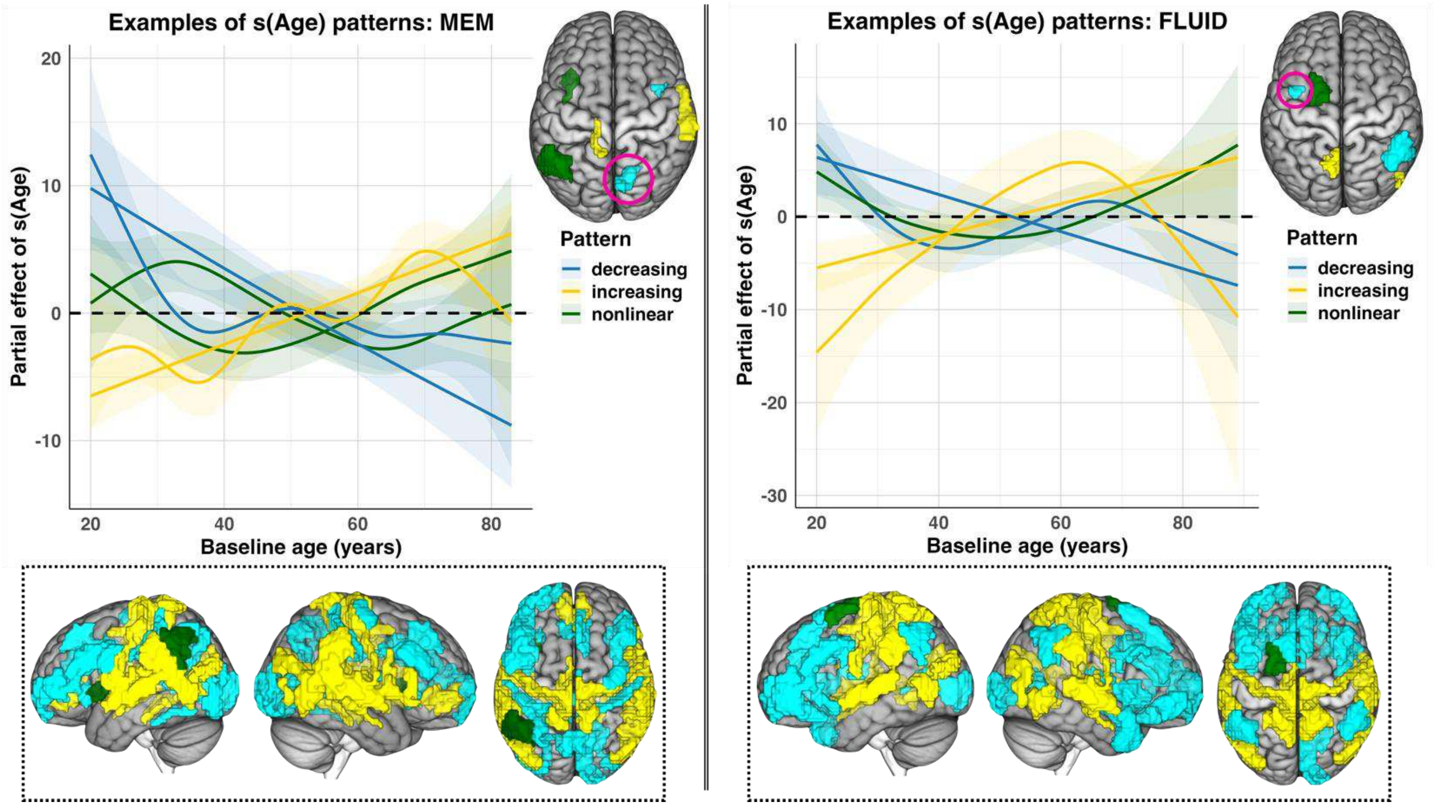

SF4. Example age-related activation trajectories (top) and cortical maps of all regions showing significant s(Age_{T0}) effects at $p < .005$ (bottom) for the MEM and FLUID domains. The exemplar curves highlight three characteristic patterns—overall age-related decreases, increases, and nonlinear/flat trajectories—with magenta circles marking regions that also exhibited significant cognition–activation coupling. Regions were not classified by edf, as edf alone does not reliably capture the direction of age effects. Instead, patterns were determined using the significance and sign of the first derivative of s(Age_{T0}), allowing differentiation between (a) trajectories that contain local wiggles but nonetheless show a consistent overall increase or decrease, and (b) genuinely nonlinear or net-flat profiles in which derivative signs fluctuate but do not yield a clear overall change across the lifespan. Regions where a significant cognition-activation coupling were found are circled in magenta.
